# Effects of Caffeinated Chewing Gum on Ice Hockey Performance after Jet Lag Intervention: Double-Blind Crossover Trial

**DOI:** 10.3390/nu16183151

**Published:** 2024-09-18

**Authors:** Ming-Tsang Tsai, Yi-Jie Shiu, Chien-Chang Ho, Che-Hsiu Chen, Chih-Hui Chiu

**Affiliations:** 1 Department of Recreational Sport, National Taiwan University of Sport, Taichung 404, Taiwan; mttsai@gm.ntus.edu.tw; 2 Department of Physical Education and Sport Sciences, National Taiwan Normal University, Taipei 106, Taiwan; shiu880511@gmail.com; 3 Department of Physical Education, Fu Jen Catholic University, New Taipei City 242, Taiwan; 093703@mail.fju.edu.tw; 4 Sports Medicine Center, Fu Jen Catholic Hospital, New Taipei City 242, Taiwan; 5 Department of Sport Performance, National Taiwan University of Sport, Taichung 404, Taiwan; jakic1114@ntus.edu.tw; 6 Graduate Program in Department of Exercise Health Science, National Taiwan University of Sport, Taichung 404, Taiwan

**Keywords:** ergogenic aids, athletes, nutrition, circadian rhythm

## Abstract

The purpose of this study was to examine the impact of caffeinated chewing gum on the physical performance of elite ice hockey players after a jet lag intervention. Fourteen national-level (age: 25.2 ± 5.4; height: 176.5 ± 5.3; weight: 78.1 ± 13.4) ice hockey players were tested late at night after a full day awake schedule with jet lag. A randomised, double-blind experimental design was employed in which participants either chewed caffeinated gum (CAF) containing 3 mg/kg caffeine or a caffeine-free placebo gum (PLA) for 10 min prior to undertaking a series of on-ice and off-ice tests. The off-ice tests included grip force, the counter-movement jump (CMJ), and the squat jump (SJ). The on-ice tests included a 35 m sprint, the S-Shape agility test, and the Yo-Yo intermittent recovery test (Yo-Yo IR1 test). The CMJ height (CAF: 47.2 ± 4.4; PL: 45.9 ± 3.5; *p* = 0.035; Cohen’s d = 0.32) and SJ height (CAF: 46.7 ± 4.1; PL: 44.9 ± 3.8; *p* = 0.047; Cohen’s d = 0.44) were found to be significantly higher in the CAF than in the PL trial. However, there were no significant differences (*p* > 0.05) in grip force, as well as in the 35 m sprint, the S-Shape agility test, and the Yo-Yo IR1 test. The present study found that, following a jet lag intervention, although the consumption of caffeinated gum resulted in an increase in vertical jump height, it had no impact on performance in the ice tests. The results of this study may help coaches and athletes consider the need for caffeine supplementation when experiencing jet lag.

## 1. Introduction

Ice hockey is one of the most popular sports in the world and has been an Olympic sport since 1920. Hockey is a high-intensity intermittent team sport. The players engage in periods of high-speed skating, interspersed with periods of passive recovery [1]. It has been demonstrated that each skating session typically lasts between 30 and 80 s, with a subsequent recovery period of approximately 2 to 5 min. Similarly, for those engaged in ice hockey, it is essential to possess the requisite strength, agility, explosive power, and aerobic endurance capacity [1]. It can be argued that, in addition to their abilities on the ice, the physical capabilities of hockey players are also a significant factor in the sport.

The influence of circadian rhythm on exercise performance has been demonstrated in numerous studies [2]. In general, the optimal time for exercise performance is usually in the evening due to the higher core body temperature, which leads to increased energy metabolism, improved muscle compliance, and the promotion of actin–myosin cross-bridging [3]. In accordance with the sleep model, the human circadian rhythm and the time since the previous period of rest both influence the characteristics of sleep [4]. It can be reasonably deduced that modifying sleep schedules and circadian rhythm times may result in enhanced training quality. However, for athletes and student athletes or when competing abroad, sleep schedules and circadian rhythms may be disrupted. Jet lag has been found to impair exercise performance in mice models and in elite athletes [5,6]. In addition to the effects of jet lag on physiological responses, studies have also demonstrated that it may have an impact on cognitive functions, including alertness, coordination, and cognitive defects [7]. It can be reasonably deduced that jet lag may have an impact on physiological rhythms, which in turn may affect athletic physiological and cognitive performance.

Caffeine is a beneficial nutrient that has been demonstrated to enhance athletic performance [8]. It has been demonstrated that caffeine supplementation prior to exercise has a significant impact on improving endurance performance and reducing the fatigue index in athletes. In sports requiring explosive power or agility, the effect size of caffeine was observed to be approximately small to moderate [9]. In order to achieve a significant boost in pre-workout strength and explosiveness in explosive sports, a higher dosage of caffeine is required [10]. However, supplementation with higher doses of caffeine may cause insomnia or affect sleep quality [11]. One potential method for addressing insomnia may be to utilise smaller doses of caffeine prior to exercise while still achieving a beneficial stimulant effect.

Caffeine gum can be absorbed through the mucous membranes of the mouth, resulting in a faster absorption rate of caffeine [12], which may also have a higher effect size on athletic performance [13]. Based on the past literature, chewing smaller doses of caffeinated chewing gum has been found to improve lower body strength and explosive performance [14]. It may be posited that the use of chewing gum as a caffeine supplement could be an effective method of enhancing athletic performance without adversely affecting sleep quality. Caffeine supplementation can be effective in improving explosive power and vertical jump height during fatigue after partial sleep deprivation [11]. However, there are no published studies investigating the impact of caffeinated chewing gum on the physical performance of ice hockey players after a jet lag intervention, whether on ice or off ice. The purpose of this study was to examine the impact of ingesting caffeinated chewing gum on the on-ice and off-ice athletic performance of hockey players after a jet lag intervention.

## 2. Materials and Methods

### 2.1. Experimental Design

This study used a randomisation crossover design with a single-blind, double-blind experimental design. The participants were randomly assigned to either a caffeinated chewing gum trial (CAF) or a placebo trial (PL). After the first main trial, participants rested and recovered for 7–10 days before proceeding to the next trial. Similar rest and recovery times have been published in past studies [13,14]. All participants completed all tests within one month. During the study period, all participants maintained their normal training status, with no changes in the training program and no over-training or extra competitions. This study was conducted during an out-of-season training period for players.

### 2.2. Participants

Fourteen trained ice hockey players (age: 25.2 ± 5.4; height: 176.5 ± 5.3; weight: 78.1 ± 13.4) were recruited to participate in this study, including six defensemen, two centres, and six forwards. All participants had more than 6 years of professional hockey training and were familiar with all hockey skills. Inclusion criteria: (i) 6 years of professional ice hockey training, (ii) 6 months of continuous training, and (iii) 3 months of recovery from sports injuries such as strains and sprains. Exclusion criteria: (i) non-specialised ice hockey player, (ii) has not trained regularly for the past 6 months, (iii) has recovered from an athletic injury, and (iv) has been recovering from a sports injury for less than 3 months or has epilepsy, hypertension, hyperlipidaemia, heart disease, arthritis, osteoporosis, brain injury, or a history of caffeine allergy. In a past study, the effect of caffeinated chewing gum was found to have an effect size (Cohen’s d) = 1.00 on the fatigue index of basketball players [13]. The G*POWER statistical software (latest ver. 3.1.9.7; Heinrich-Heine-Universität Düsseldorf, Düsseldorf, Germany) indicated that eight participants were sufficient to interpret the data for the purposes of this study, with a power value of 0.8. In order to achieve a more precise POWER value, the number of participants in this study was set to 14. All participants were provided written consent forms after being fully informed about the potential risks involved in the experiment, in accordance with the ethical standards of the relevant academic institution. A signed consent form was obtained from each participant. The order of participants was determined through the use of computerised randomisation software (Microsoft software package, Excel 365, subscription 2017)). This study received approval from the Institutional Review Board of Jen-Ai Hospital-Dali Branch (202300071B0; date: 31 October 2023) and was registered at ClinicalTrials.gov (date: 16 August 2024; ID “NCT06557655”; https://register.clinicaltrials.gov). All data were collected at an indoor stadium. This study was conducted under the Declaration of Helsinki.

### 2.3. Protocol

All participants were required to perform the exercise-specific tests at least 2 times prior to the formal trial to familiarise themselves with the process of the formal trial. Participants were asked to record their diet 3 days before the first formal trial and were asked to repeat the same diet before the next trial. The following experiments were scheduled to start at 11 p.m. Upon arrival at the designated ice hockey arena, the participants were granted a 10 min rest.

All participants remained awake (CAF: 11.5 ± 2.2 h; PL: 11.8 ± 2.2 h) for a full day after adequate sleep (CAF: 7.4 ± 1.4 h; PL: 7.1 ± 1.2 h), with no nap allowed in between. Participants were also asked to be exposed to sunlight or lights as much as possible during the day to avoid darkness. Such a method has been shown in previous studies to induce jet lag and decrease exercise performance [5]. After 10 min of break, participants chewed either caffeinated chewing gum (CAF) containing 3 mg/kg of caffeine or caffeine-free chewing gum (PL) for 10 min each time. After spitting out the chewing gum, participants performed dynamic stretching and a warm-up. After the warm-up, a 1 min break was taken, followed by both off-ice and on-ice tests. The consumption of caffeinated chewing gum at the specified dosage and timing has been shown to significantly enhance sprinting speed, increase lower body strength, and reduce anaerobic fatigue index in basketball players [13]. The measures employed in this study can be classified into two principal categories: off-ice tests and on-ice tests. In previous studies, a significant positive correlation has been identified between the off-ice tests and on-ice tests [15]. Accordingly, the present study distinguished between off-ice and on-ice tests. The off-ice tests include grip force, counter-movement jump, and squat jump [15]. The on-ice tests include 35 m sprint [15], S-Shape agility test [16], and Yo-Yo intermittent recovery test on ice [17]. Participants were first tested on land, followed by a 10 min break, and then put on ice hockey equipment to be tested on ice.

### 2.4. Outcome Measures

The counter-movement jump was measured using Gymaware (GymAware, KineticPerformance, Canberra, Australia). The device strap was secured to the participant’s waistband, and they were instructed to remain as still as possible and place their hands on their waist. They were then required to squat down to the ground in a parallel position with their thighs and then jump up as quickly as possible. The experimental procedure was conducted twice, with a one-minute interval between trials. The mean value of the two trials was calculated as the final result.

The squat jump was measured using Gymaware. The device strap should be secured to the participant’s waistband. The participant is instructed to remain as still as possible and place their hands on their waist. They are then required to squat down to the ground in a parallel position with their thighs for 2–3 s and then jump up. The experimental procedure was conducted twice, with a one-minute interval between trials. The mean value of the two trials was calculated as the final result. The same vertical jump test has been employed in a previous study [13].

In the grip force test, the participant stood in a standing position holding a grip dynamometer (Smedlay’s Hand Grip Dynamometer TTM, Tokyo, Japan) [18], with the grip adjusted to the second knuckle and the arm at about 10–15 degrees to the trunk, and continuously pressed the grip for about 3 s at maximum force. Both hands were tested twice with a one-minute rest in between. The average strength of both hands was taken as the result.

The 35 m sprint on ice [15] was measured using a timing grating (Witty, Microgate, Bolzano, Italy) with an accuracy of 0.01 s. The first timing grating was placed at the starting position, and the second timing grating was placed at the 35 m position. Participants dressed in full ice hockey equipment, remained stationary, held the stick, and retreated behind the starting line. They began to complete the 35 m sprint with maximum effort, keeping the stick close to the ice surface during the process. Each experiment was performed twice, with a three-minute break in the middle of the experiment, to achieve the best results.

The ice S-Shape agility test was conducted using a timing grating (Witty, Microgate, Bolzano, Italy). The participants dressed in full hockey equipment and held a hockey stick. They stood behind the goal line and skated along the outside edge of two face-off circles in a figure-of-8 formation without touching the face-off circles, then sprinted in a straight line across the near blue line of the rink with the stick close to the ice surface. This approach has been used in the literature in the past [16]. Each experiment was performed twice with a three-minute break in between, and the best score was taken.

The on-ice Yo-Yo IR test measures the aerobic endurance of hockey players [17]. Cones were placed at the start point, the finish point (20 m), and the buffer zone (5 m after the start point). Participants stood on ice with full hockey equipment and a hockey stick. A 20 m sprint was performed after hearing an indicator tone, with a 10 s rest period after every 2 sprints. During the test, the indicator tone would speed up over time. During the process, players who did not reach the opposite cone before the indicator tone two consecutive times failed the test, and the results of the previous stage were recorded.

### 2.5. Caffeine and Placebo Gum

The caffeinated chewing gum used in this study, specifically Military Energy Gum (Arctic Mint flavor; Stay Alert, Chicago, IL, USA), has been previously utilised in other studies [14,19]. Each piece contained 100 mg of caffeine, with a 5 g gum base. The gum was provided in various weights, according to the relative dosage. A commercially available blue mint gum was used as the placebo. To achieve a target dose of 3 mg caffeine per kilogram of body weight and maintain a double-blind design, all chewing gums were mashed, ground, homogenised, and reshaped after incorporating 0.3 g of peppermint flavour powder. This ensured that the colour, appearance, taste, weight, and size of the gum types were indistinguishable. All chewing gums were prepared by a specialised individual and given to the on-site testers after numbering.

### 2.6. Statistical Analysis

All data are presented as means ± standard deviations. The normality of the data distribution was assessed using the Shapiro–Wilk test. The grip force, CMJ, SJ, 35 m sprint, S-Shape agility test, and Yo-Yo intermittent recovery test were analysed through paired *t*-tests. The magnitude of the observed effects was quantified using Cohen’s d, a measure of effect size, and defined as trivial (<0.20), small (≥0.20–0.59), moderate (≥0.60–1.19), large (≥1.20–1.99), and very large (≥2.00) [20]. The power value of each data point was evaluated using G*Power 3.1.9.6 software [21]. All data were calculated using SPSS (version 20, Chicago, IL, USA), and the significance level was *p* < 0.05.

## 3. Results

### 3.1. Baseline Parameters

There were no significant differences in the pre-test sleep duration, wakefulness, and RPE between the two trials (Table 1).

### 3.2. Off-Ice Tests

**Figure 1:**
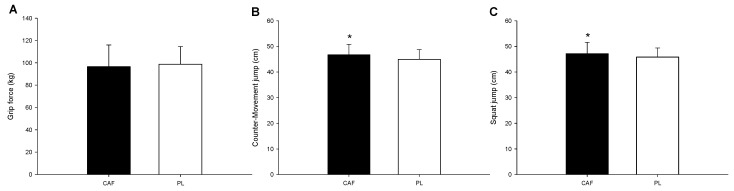
Off-ice tests. The grip force (**A**), jump height of counter-movement jump (**B**), and jump height of squat jump (**C**). * CAF was significantly different to PL.

There was no significant difference in the grip force (Figure 1A) between the two trials (*p* = 0.412). However, small significant differences were found in the jump height, with CAF displaying higher values than PL (CAF: 47.2 ± 4.4; PL: 45.9 ± 3.5; *p* = 0.035; Cohen’s d = 0.32) in the CMJ (Figure 1B) and the SJ (CAF: 46.7 ± 4.1; PL: 44.9 ± 3.8; *p* = 0.047; Cohen’s d = 0.44) (Figure 1C).

### 3.3. On-Ice Tests

**Figure 2:**
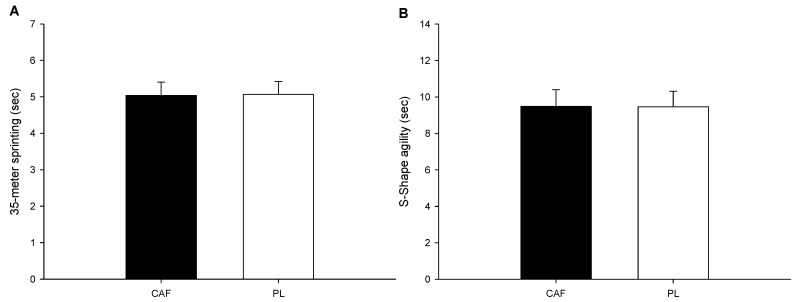
On-ice tests. The 35 m sprint (**A**) and S-Shape agility test (**B**).

The results of the 35 m sprint on ice and S-Shape agility test between the two trials are presented in Figure 2A,B. The results of this study showed that there was no significant difference between the two groups in the 35 m sprint on ice (*p* = 0.455; Cohen’s d = 0.27) and the S-Shape agility test (*p* = 0.64; Cohen’s d = 0.13).

### 3.4. On-Ice Yo-Yo IR1 Test

**Figure 3:**
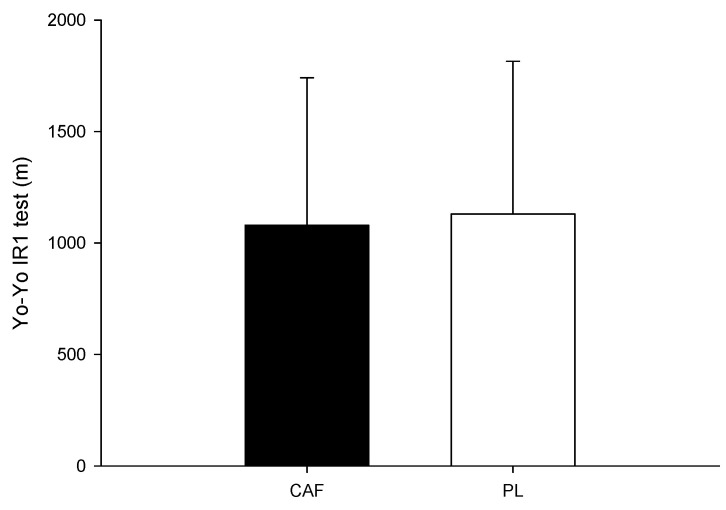
On-ice Yo-Yo IR1 test.

No significant difference was found in the distance of the Yo-Yo IR1 test (Figure 3) between the two trials (*p* = 0.779; Cohen’s d = 0.07).

## 4. Discussion

The purpose of this study was to examine the impact of ingesting caffeinated chewing gum on the on-ice and off-ice athletic performance of hockey players after a jet lag intervention. The present study found that supplementation with caffeinated chewing gum after a jet lag intervention was effective in improving the CMJ and SJ heights. However, the improved vertical jump height was not actually reflected in the on-ice sprinting and agility tests. On the other hand, chewing caffeinated gum did not improve aerobic endurance performance on ice.

The efficacy of caffeine supplementation in enhancing athletic performance among hockey players remains inconclusive, with findings from various studies yielding contradictory results. A study conducted by Madden et al. (2019) revealed that the supplementation of caffeine led to a significant enhancement in sprint speed among hockey players. However, no impact was observed on hockey-specific athletic ability [22]. In another crossover double-blind study, supplementation with 3mg of caffeine per kilogram of body weight was found to have no effect on hockey players’ sprinting or agility [23]. In contrast to the results of previous studies, the present study, which involved top national-level ice hockey players chewing caffeinated gum containing 3 mg/kg caffeine while under a jet lag intervention, demonstrated efficacy in enhancing the vertical jump heights of the hockey players. However, the enhanced explosive power was not reflected in the on-ice sprinting and agility. It was therefore proposed that caffeine supplementation combined with a jet lag intervention would not enhance the on-ice performance of national-team-level-trained ice hockey players.

The sport of ice hockey demands a high level of on-ice motor control, cognitive function, and skating skills [24]. Although previous studies have identified a significant correlation between vertical jump height and on-ice skills [15], the question of the causal relationship between enhanced vertical jump height and enhanced on-ice sprinting ability remains unanswered. Indeed, the existing literature indicates that caffeine supplementation exerts an influence on physical performance yet no discernible impact on the specific skill set required for hockey [22]. In addition, some studies have found that measurements on land do not reflect measurements on ice [25]. The discrepancy between vertical jump height and on-ice ability may be attributed to the variations in stride length and differences in motor unit recruitment, making it impossible to directly reflect on-ice performance in off-ice tests [26]. In this study, the coefficient of variation (CV) was 6.4 and 6.5% for the 35 m sprint and 9.2 and 8.7% for the J-shape test, indicating that the participants recruited for this study had reached a considerable level of consistency in their on-ice ability. The data from this study clearly demonstrated that, under the conditions of a jet lag intervention, the ingestion of caffeine gum, while improving vertical jump height, did not correspond with an improvement in athleticism on the ice.

Although caffeine has been found to be effective in improving endurance exercise performance in many publications [9], the present study used the Yo-Yo IR1 test on ice as an indicator of aerobic capacity and found no significant difference in aerobic capacity between the two trials after chewing caffeinated gum. There are several reasons for this result. First, this study was conducted during the off season and included forwards, centres, and defensemen. During the off season, many athletes focus on weight training, resulting in greater variation in aerobic capacity between individuals. The second possible reason is that jet lag causes a decline in aerobic capacity [27]. In past studies, caffeine supplementation after partial sleep deprivation was found to have no effect on the ability to repetitively sprint [28]. However, there is still a lack of relevant studies examining this topic as to whether there is an effect on aerobic capacity. Therefore, the data of this study depicted that chewing caffeinated gum after a jet lag intervention did not manage to significantly affect the aerobic performance of hockey players.

The strength of this research is that the effects of the consumption of caffeinated chewing gum on the hockey performance of elite hockey players after a jet lag intervention were studied. The present study found that even though the vertical jump heights tested on off-ice tests increased, this did not necessarily reflect the ability tested on the ice. This approach is more consistent with the physiological state of the athlete prior to training or competition. Despite the rigorous methodology employed to control for the study variables, this study was subject to several limitations. It should be noted that this study did not analyse the caffeine concentration in either the blood or saliva. Nevertheless, previous research has demonstrated that the ingestion of an equivalent quantity of chewing gum at the same time has been shown to effectively elevate caffeine levels in the blood plasma [12]. Consequently, we believe that the methodology employed in this study remains a viable approach. Secondly, we only recorded the sleep time of the previous day and did not monitor the sleep quality of the participants through other instruments. However, in terms of the RPE values, the RPE values before the start of the two experiments were similar, indicating that the participants had similar levels of fatigue. In addition, we also strictly controlled the diet of the participants and their exposure to light during the jet lag period. In this case, chewing caffeinated gum did not improve the on-ice athletic performance of hockey players.

## 5. Conclusions

The present study found that chewing caffeine-containing gum after a jet lag intervention significantly improved CMJ and SJ heights. However, the improved vertical jump height was not actually reflected in on-ice sprinting and agility measures. On the other hand, chewing caffeinated gum also failed to improve on-ice aerobic endurance performance. The results of this study can be used as a reference for coaches and athletes in preparation for competitions. The measurement of vertical jump height prior to participation in ice hockey does not constitute a reliable indicator of an individual’s direct on-ice ability. When an athlete is affected by jet lag, the use of caffeine may increase explosive power but may not affect motor mechanisms or cognitive functions, resulting in no improvement in ice test scores.

## Figures and Tables

**Table 1:** Baseline parameters.

	CAF	PL	*p*
Sleep time (hours)	7.4 ± 1.4	7.1 ± 1.2	0.231
Awake time (hours)	11.5 ± 2.2	11.8 ± 2.2	0.261
RPE before test	5.7 ± 1.9	6.0 ± 1.7	0.598

Values are mean SD, n = 19. CAF, caffeine trial; PL; RPE, rating of perceived exertion.

## Data Availability

All relevant materials are presented in the present manuscript.
